# An experimental method to investigate the water-based suppression of smoldering peat fire

**DOI:** 10.1016/j.mex.2020.100934

**Published:** 2020-05-28

**Authors:** Shaorun Lin, Xinyan Huang

**Affiliations:** a Research Centre for Fire Engineering, Department of Building Services Engineering, The Hong Kong Polytechnic University, Kowloon, Hong Kong SAR; b The Hong Kong Polytechnic University Shenzhen Research Institute, Shenzhen, China

**Keywords:** Water spray, Underground fire, Fire suppression, Rain suppression, Lab fire test

## Abstract

Smoldering wildfire in peatlands is one of the largest and most persistent fire phenomena on Earth, which contributes importantly to global carbon emissions. However, very few studies are available on how to extinguish these smoldering wildfires. Herein, we develop an experimental method to explore the effectiveness of the water-based suppression on smoldering peat fire, and the proposed method can also be used to simulate fire suppression by rain. The low-temperature drying process avoids the variation of hydrophobicity of peat soil. The validation shows that the proposed approach can provide a wide range of water-spray intensities with time variation, and two examples of successful suppression of smoldering peat fire and flaming wood crib fire were presented.

• The design of small-scale combustion reactor for peat can mimic the smoldering peat fire in the field.
• The effectiveness of water-based fire suppression technologies on peat fire are explored in the lab.
• The proposed method can evaluate the effect of rain and weather on suppressing the smoldering wildfire.

## Graphical Abstract

**Figure:**
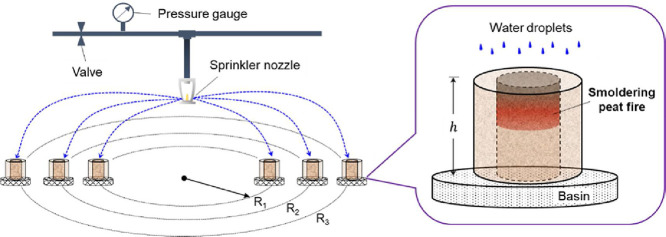

**Table:** Specifications Table

Subject Area	Environmental Science
More specific subject area	*Wildfire suppression*
Method name	*An experimental method to investigate the water-based suppression of smoldering peat fire*
Name and reference of original method	*Lin et al. (2020) Can rain suppress smoldering peat fire? Sci. Total Environ.*https://doi.org/10.1016/j.scitotenv.2020.138468
Resource availability	*N/A*

## Method details

### Collection and preprocessing of peat soil

The peat soil, a typical wildland fuel that supports smoldering fire, was tested in this work [1], which can be collected from the field or purchased from the market. The peat with uniform density, particle size, organic content has better repeatability of the fire experiments. Thus, the commercial peat soil is recommended due to its consistency and availability [2].

Moisture content (MC) is one of the most important parameters that govern the ignitability of peat soil [3], [4], [5]. In order to control the MC of peat sample and simulate a natural drying process [3,6,7], the peat sample was dried in an oven with a constant and uniform temperature of 40 °C. Such an oven temperature was close to the ambient temperature in the tropical regions in the dry season [7]. During the drying process, the weight and moisture of peat were monitored every 1 h until its MC was close to the targeted value (e.g., 50%). Herein, the high-temperature heating is not recommended since it will increase the hydrophobicity of peat soil and even affect its physicochemical properties [8]. Afterward, the sample were shaken to improve the mixing process and left in a sealed box for homogenization for at least another 48 h [9]. Before the experiment, the MC of peat sample should be further checked.

### Ignition of peat fire

**Fig. 1:**
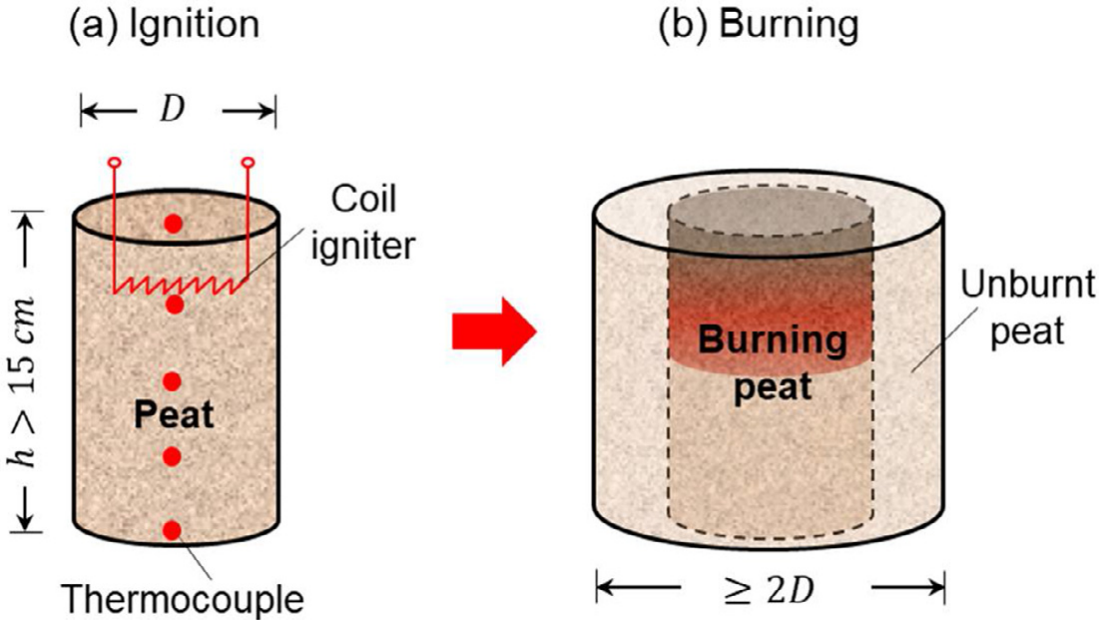
The schematics of (a) ignition and (b) self-stabilization burning process of peat soil [1].

The peat sample was filled in a cylindrical mesh reactor, as shown in Fig. 1. In general, the diameter of the reactor (*D*) should be larger than 10 cm. Otherwise, the large surface-to-volume ratio for smaller sample will result in a larger heat loss as well as a lower smoldering temperature and fire spread rate [2,10], which substantially deviates from the field condition. On the other hand, in order to study the water-based suppression of smoldering peat fire at different depths, the height of the reactor (*h*) should be larger than 15 cm. Note that the height and the diameter should be varied simultaneously to ensure sufficient oxygen supply in the deeper layers [9,11]. Generally, the height-to-diameter ratio should at least not be smaller than 1:1.

The cylindrical basket was made of steel mesh to ensure good oxygen supply to peat fire and minimize the influence on water flow. After filling in, the peat sample should be compressed slightly to ensure the dry bulk density of peat is fixed (e.g. 145 kg/m^3^ in [1]) regardless of the MC, which is closer to its natural condition [3]. To study the water-based suppression on both the shallow and deep underground peat fire, a long coil igniter (Cr_20_Ni_80_) was placed at different depths below the top free sample surface to start the smoldering peat fire. Therefore, both downward [9] and upward [11] smoldering fire spread, as well as the shallow and deep smoldering fire at different depths can be studied. The ignition should be strong enough to initiate a robust smoldering fire, e.g., 60 W for 60 min or 100 W for 30 min, depending on the peat conditions [1,7,9].

Unlike the visible flaming fire, it is difficult to judge the effectiveness and success of suppressing the smoldering fire, especially in the deep soil layer. Therefore, an array of armored K-type thermocouples with 1-mm bead diameter were inserted into the peat sample at different depths to monitor the temperature and the location of the smoldering front [9,10]. In general, the interval between the thermocouples should not be lower than 2 cm and be larger than 5 cm. After ignition, the basket of peat sample was placed into a larger cylindrical mesh basket with a diameter of  > 2*D* and a height of *h*. In order to simulate the natural state and mimic a real boundary condition, the space between two baskets was filled with unheated peat soils [1]. Note that the coil igniter will not be removed after the ignition so as not to damage the upper structure of the sample. Afterward, the entire setup was left to burn and self-stabilize for another 30 min before the start of water suppression.

### Simulation of rain

**Fig. 2:**
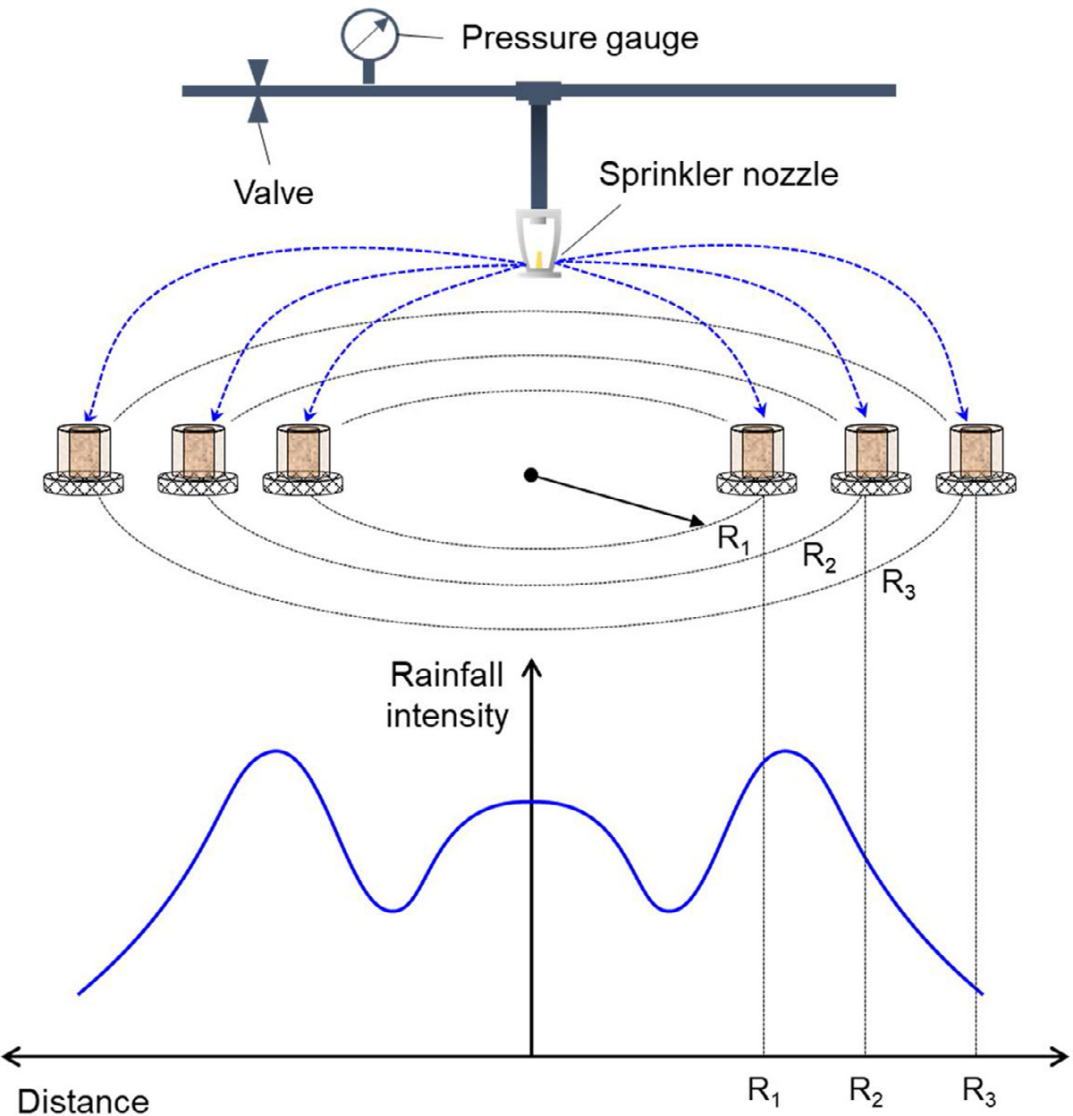
Illustration of water-based suppression simulated by a sprinkler system [1].

The water-based suppression experiments were conducted in a large wet chamber (30 m^2^) with relatively constant ambient temperature (23°C), relatively humidity (50%) and ambient pressure (1 atm). The water droplet was generated by a water sprinkler system that includes a sprinkler nozzle, a pressure gauge, and a valve, as illustrated in Fig. 2 and Video S1. In other words, the height between water spray and fire is large (> 2 m), which is different from the short-distance water spray in [12]. The larger distance between sprinkler nozzle and burning fuels increases the uniformity of water spray, better than previous work. In addition, a fan can be installed in the wet chamber to mimic various wind conditions in the field, but the wind could also change both the fire behavior and the rainfall distribution. Nevertheless, wind only has a weak effect on the underground smoldering peat fire, so it is not considered in this work.

The height between the nozzle and the sample surface can also be adjusted. The water droplet size depends on the water pressure (*P*), sprinkler orifice diameter (*D*), and the surface tension of the air-water interface (σ=0.073N/m). For example, an empirical correlation can be used to estimate the median water droplet diameter (*d_m_*) as

(1) $$d_{m}=C\frac{D^{2/3}\sigma^{1/3}}{P^{1/3}}$$

where *C* is an empirical constant for a specific sprinkler nozzle [13]. Therefore, it is possible to explore the influence of water droplet size on the fire suppression by varying the type of nozzle and water pressure. Like the rain drop, large water droplets will break into smaller ones when their surface tension cannot overcome the air drag.

The intensity of water spray changed with the location, therefore, this method can also be used to simulate fire suppression by rain with different rainfall intensities [1]. The distribution of water spray was measured by multiple cylindrical containers (with the same diameter and height of the cylindrical reactor) at different locations. For example, given a fixed duration (Δ*t*) of 15 min, the local water spray intensities (*I*) can be calculated by measuring the height of water in the containers (*d*) as I=d/Δt. Then, the desired water spray intensities can be achieved by placing the burning sample at a specific location. Moreover, the time-dependent rainfall intensity can also be simulated by either changing the pressure of water pressure (*P*) with time or moving the location of fuel sample (*R*).

### Experimental procedure

After igniting the peat by the coil heater for 60 min, the power supply was turned off. The peat should be left burning for another 30 min for stabilizing and confirming the successful ignition. Then, the peat sample was positioned to the prescribed location, and the sprinkler system was activated for a prescribed duration. Afterward, the peat sample was left for another 24 h to determine whether the smoldering fire can still survive to burnout the peat. Unlike the suppression of flaming fire, it was not possible to instantaneously and visually determine whether the smoldering peat fire was extinguished or not. If the temperatures inside the sample re-rose above 250°C, i.e., the minimum smoldering temperature of peat [3], and if the peat sample eventually burned out, the fire-suppression was considered as a failure. Then, a repeating test should be conducted to confirm the outcome. Afterward, experiments were continued under a longer water-spray duration with fresh peat samples until critical conditions (water-spray duration and depth) of suppression are found.

### Reference experiments (flaming fire suppression)

The proposed experimental setup and procedure is equally suitable for the water-based suppression of flaming fire and testing different sprinkler and water-mist systems. In literature, the wood crib fires were widely used as a standard flaming fire [14]; thus it is also recommended as a reference experiment. To enforce a fair comparison, the burning area of the wood crib should be similar to the smoldering peat fire. The wood cribs were ignited by a lighter for 1 min, followed by 1 min of self-burning before the water-based suppression. The extinguishing limit of the flaming wood crib was determined in the same way as those of the smoldering peat fire.

## Method validation

### Distribution of water-spray intensity

**Fig. 3:**
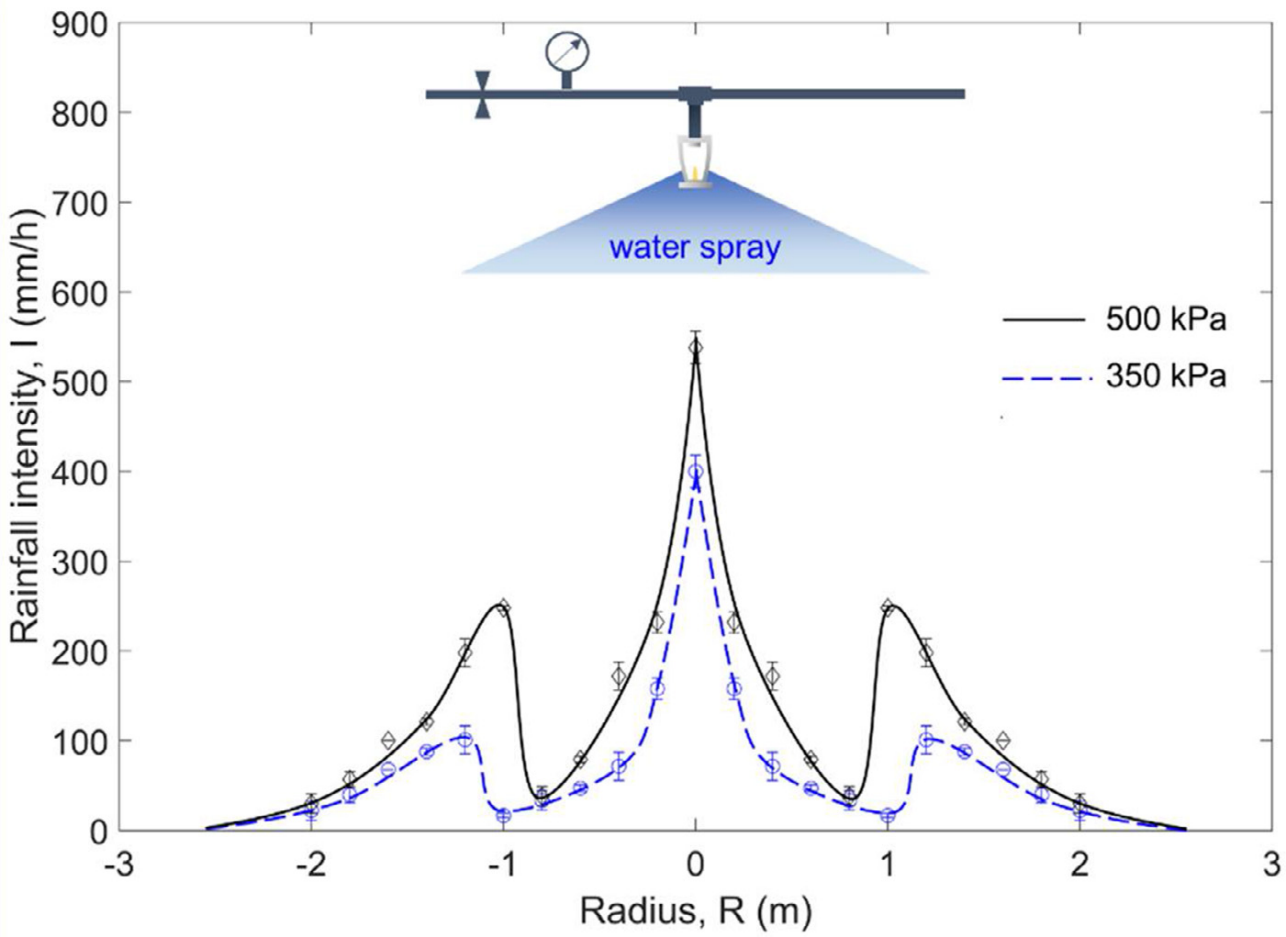
Measured distribution of the water-spray intensity from a sprinkler system at different water pressures, where the symbols represent the average values, and error bars represent the standard deviation.

The proposed method was used to simulate the effect of rain (a natural water-based suppression) on suppressing smoldering and flaming wildfires. The water pressure *P* was set to about 500 kPa, the sprinkler orifice diameter *D* was 15 mm, and the surface tension of air-water interface δ is 0.073 N/m. according to the Eq. 1, the median water droplet diameter (*d_m_*) was calculated as around 1 mm, which was within the range of typical raindrop sizes [15]. By putting multiple cylindrical containers at different positions, the distribution of water-spray (or simulated rainfall) intensity can be measured. For example, Fig. 3 shows the distribution of rainfall intensities at the water pressure of 350 kPa (~50 psi) and 500 kPa (~70 psi), where the standard deviation is small. Therefore, a wide range of rainfall intensities can be achieved and varied with time by changing the pressure of water pressure or moving the fuel sample.

### Water-based suppression of smoldering peat fire

**Fig. 4:**
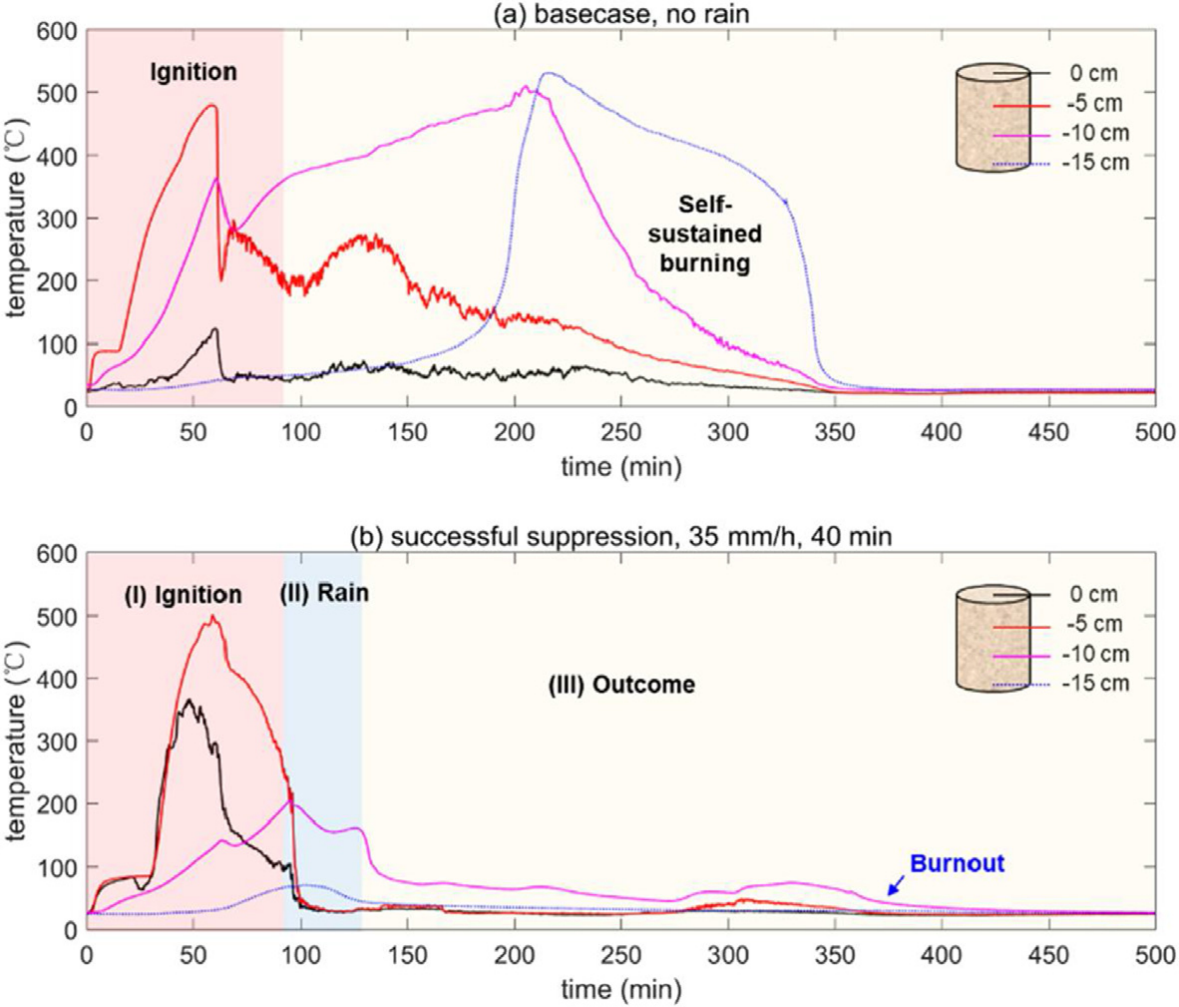
Temperature measurement of (a) base case without rain suppression, and (b) successful suppression with rainfall intensity of 35 mm/h for 40 min.

For smoldering peat fire, the cylindrical reactor we used had a diameter of 10 cm and a height of 15 cm. And four thermocouples were inserted into the peat sample at different depths at 0 cm (surface), 5 cm, 10 cm and 15 cm to monitor the temperature. The coil heater was buried 5 cm below the top free surface, and the ignition protocol was 60 W for 60 min. Fig. 4a shows a group of thermocouple measurements of the baseline experiment without suppression, and a typical smoldering spread over a 15-cm deep peat sample can last for about 350 min (about 6 h).

Fig. 4b shows an example of the temperature evolution inside the smoldering peat soil under a rainfall intensity of 35 mm/h for 40 min, and the fire suppression was successful (see Video S1). Once the simulated rain was activated, the temperature of the upper layer decreased dramatically to the ambient temperature, while the temperatures at the deeper layers showed a smaller decrease, because it took time for water to penetrate into soil. After suppressing for 40 min, despite some fluctuations, all temperatures gradually decreased to ambient temperature, revealing a successful suppression of smoldering peat fire.

### Suppression of flaming wood-crib fire

**Fig. 5:**
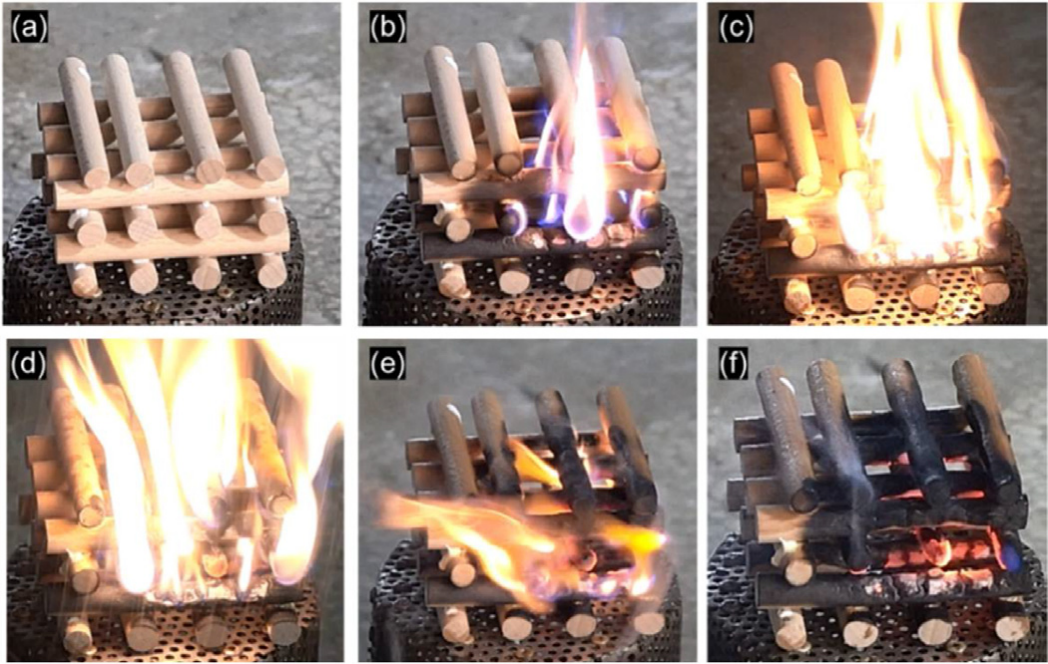
Photos of fire-suppression procedure of flaming wood crib fire. (a) Before ignition, (b) right after ignition, (c) growth of flame, (d) right before rain suppression, (e) during rain suppression, and (d) extinction [1].

For the suppression of flaming fire, the wood crib was chosen to mimic the common flaming fire on twigs [1]. The wood crib was made of cylindrical wood rods with a length of 8 cm and a diameter of 1 cm [16], whose area was similar to the smoldering peat fire (Figure 5), thus, ensuring a fair comparison [1]. The ignition lasted for 1 min, and self-sustained burning lasted for another 1 min. Then, the water spray (or simulated rain) was activated. Unlike smoldering fire, it is easy to judge the suppression effect of flaming fire visually. After the suppression for about 40 s, the flame was extinguished, revealing a successful suppression of flaming fire.

## Declaration of competing interests

The authors declare that they have no known competing financial interests or personal relationships that could have appeared to influence the work reported in this paper.

## Appendix Supplementary materials

Video S1. Suppression of smoldering peat fire by simulated rain. Image, video 1
